# Supplementary material for: Nucleoside binding by a surface lipoprotein governs conjugative ICE acquisition in mycoplasmas
Source: mBio. 2025 Dec 30;17(2):e02939-25. doi: 10.1128/mbio.02939-25 (PMC12892991; doi:10.1128/mbio.02939-25)
Supplement: Supplemental Figures — Fig. S1 to S7. [file mbio.02939-25-s0001.pdf]

```
RM16 (FWK06 _000085)    MKKNKFYLFLGAAPVLSVPLVAASCGDKYFKETEVDGVKTVTTLSHIVSRKGLKLRDGLT
PG2 (MAG0120)           MKKNKFYLFLGAAPVLSVPLVAASCGDKYFKETEVDGVKTISTLAHITSRKGLKLREGLT
5632 (MAGa0140)         MKKNKFYLFLGAAPVLSVPLVAASCGDKYFKETEVDGVKTISTLAHITSRKGLKLREGLT
                        ****************************************::**:**.********:***

RM16 (FWK06 _000085)    VDNAPRAAFITDEGSVHDESFNQSGWEAVHKISYELGLDKAQVSGNKNLRNKVYEPKKGE
PG2 (MAG0120)           VENAPKATFITDEGSVHDESFNQSGWEAVHKVSYELGLDKAQVSGNKNLRNKVYEPKKGQ
5632 (MAGa0140)         VENAPKATFITDEGSVHDESFNQSGWEAVHKVSYELGLDKAQVSGNKNLRNKVYEPKKGQ
                        *:***.*:***********************:***************************:

RM16 (FWK06 _000085)    LASSYKNAIDSSFRYIVLCGFTHKAALYGLEPEYIKKIKDNNIVFITVDFDIQQDASTGE
PG2 (MAG0120)           LLEAYKNAIDSGFRYIVLCGFTHQASLVGLDENYIKKIKDNNIIFITVDFNLFTE---DD
5632 (MAGa0140)         LLEAYKNAIDSGFKYIVLCGFTHQAALVGLDANYIKKIKDNNIVFITVDFDLFTE---ND
                        * .:*******.*.*********:*:* **: :**********:******::  :   .:

RM16 (FWK06 _000085)    PAAKAFVDKIGQGRLIPVIFDTKQAAYIAGRALADYFSKIYKDNPEKRTIGAFGGIPWPA
PG2 (MAG0120)           ANVKTFIKKIGEGHLVPVIFDTKQAAYIAGRALADYFSQVYKDQPEKRTIGAFGGIPWPA
5632 (MAGa0140)         ANVKTFIEKIGEGHLVPVIFDTKQAAYIAGRALADYFSQVYKDQPEKRTIGAFGGIPYPA
                        . .*:*:.***:*.*:**********************::***:*************:**

RM16 (FWK06 _000085)    VSDFIAGTFQGIIDWNKEHPEAKTKSLNNTIELKTSFTSGEPVAVAAINSVIKATASYPV
PG2 (MAG0120)           VSDFIAGTFQGIIDWNKEHPEAKTKSLNETIELNTLFTSGTPQATTAINSVVKATASYPV
5632 (MAGa0140)         VSDFIAGTFQGIIDWNKEHPDVKTKSLNETIELNTLFTSGTPQATTAINSVVKATASYPV
                        ********************:.******:****:* **** * *.:*****:********

RM16 (FWK06 _000085)    AGSLSSDTAKEIKKLGDKNKFIIGVDADQKNALKGHRIFTSVMKLIGQAVYNVLADLYSQ
PG2 (MAG0120)           AGSLSTDTAKEIKKLADKDKFIIGVDADQKNALKGHRIFTSVMKLIGQAVYNILADLYSK
5632 (MAGa0140)         AGSLSTDTAKEIKKLADKDKFIIGVDADQKNALKGHRIFTSVMKLIGQAVYNILADLYSK
                        *****:*********.**:*********************************:******:

RM16 (FWK06 _000085)    GENSLSLQPGFEIGKKNGEAKVFGYGENEASKYVGVATSGLLDSKNDEIANKALEEATKY
PG2 (MAG0120)           GENQLDLQPGFEIGKKNGTPTVFGYGDTEDKQYVGVATSGLLDDKNDEIANKALKDATAY
5632 (MAGa0140)         GENQLDLQSGFEIGKKNGIPTVFGYGDTEDKQYVGVATSGLLDNKNDEIANKALKDATAY
                        ***.*.**.********* ..*****:.* .:***********.**********::** *

RM16 (FWK06 _000085)    YESKKAEIQKTLSGQLEEAKKALGTKWPDQPADQFGKMINWLAKETQK
PG2 (MAG0120)           YVQKKTEIQKSLKDQMETAKKALGAKFPSDPGGQFQKMVDWLASETRK
5632 (MAGa0140)         YVQKKADIQKSLKDQIETAKKALGNKFPSDPGGQFQKMVDWLASETRK
                        * .**::***:*..*:* ****** *:*.:*..** **::***.**.*
```

Figure S1. Multiple sequence alignment of the P48 lipoprotein from *M. agalactiae* PG2 and its homologs from *M. agalactiae* 5632 and *M. bovis* RM16 strains. Multiple sequence alignment by MUSCLE (3.8). CDS accession numbers in the GenBank database are indicated in parentheses.

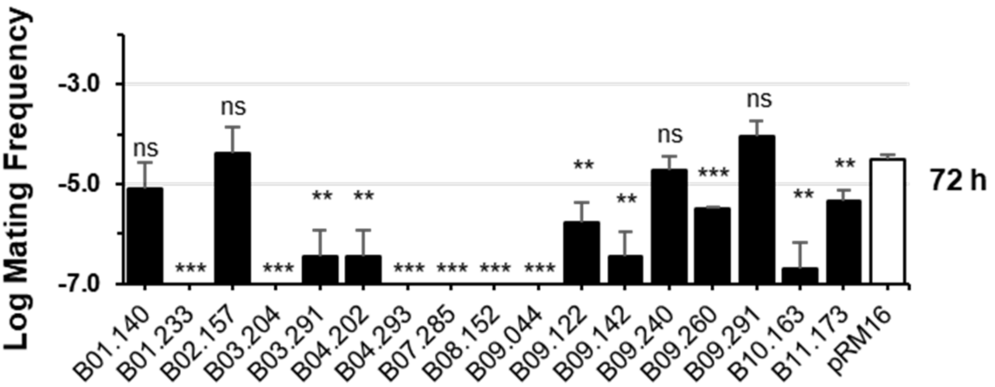

Figure S2. Resistance of *M. bovis* mutants to ICE transfer. ICE transfer from J228[ICEB]^{G} was measured to assess resistance. The recipient pRM16^{P} (pRM16) was used as positive controls (Table 1). Mating frequency was calculated as the ratio of dual-resistant transconjugants to total CFU. Data represent the mean ± SD of ≥3 independent experiments. Statistical significance was determined using two-tailed t-tests relative to pRM16^{P}: ns, $p \geq 0.05$; **, $p < 0.01$; ***, $p < 0.001$.

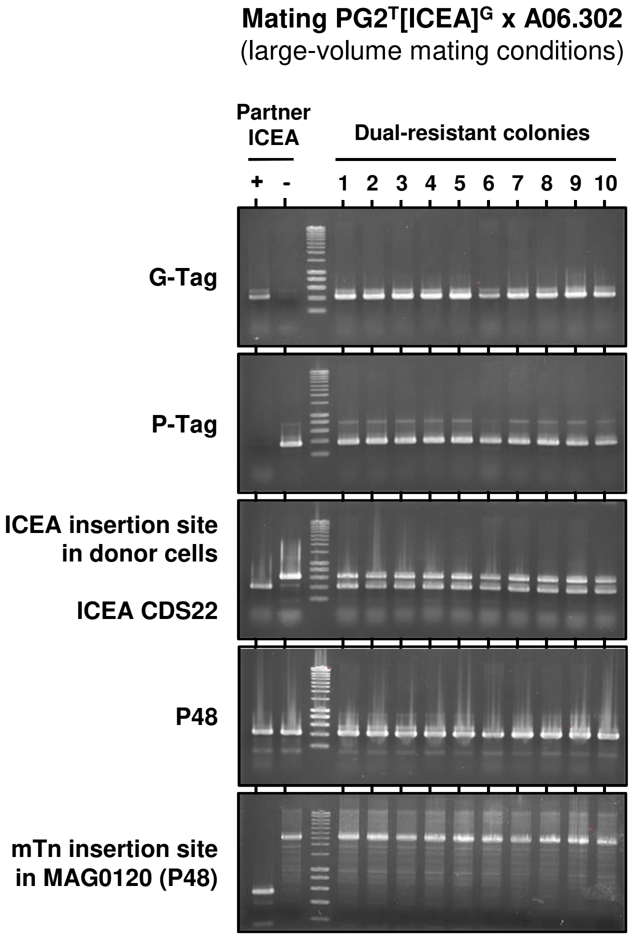

Figure S3. PCR characterization of dual-resistant colonies from mating PG2^{T}[ICEA]^{G} x A06.302. PCR amplification of 10 dual-resistant colonies selected from large-volume mating conditions. All dual-resistant colonies were confirmed as transconjugants by PCR detection of antibiotic resistance markers originating from the ICEA donor PG2^{T}[ICEA]^{G} (G-Tag) and from the ICEA recipient A06.302 (P-Tag). They were also tested positive for the ICEA CDS22 (ICEA CDS22) and the chromosomal region flanking the ICEA insertion site in the donor (ICEA insertion site in donor cells), indicating that in these transconjugants, the ICEA is integrated at a different chromosomal location. Additionally, the transconjugants were PCR positive for the *p48* gene MAG0120 (P48). However, PCR amplification of the region spanning the mTn insertion site in the *p48* mutant A06.302 (mTn insertion site in MAG0120) yielded a large amplicon (> 2.5 Kb), confirming that the transconjugants are *p48* mutants. The oligonucleotide primers used for PCR amplifications are described in Table S1. Mating partners were used as controls (ICEA+, PG2^{T}[ICEA]^{G}; ICEA-, A06.302).

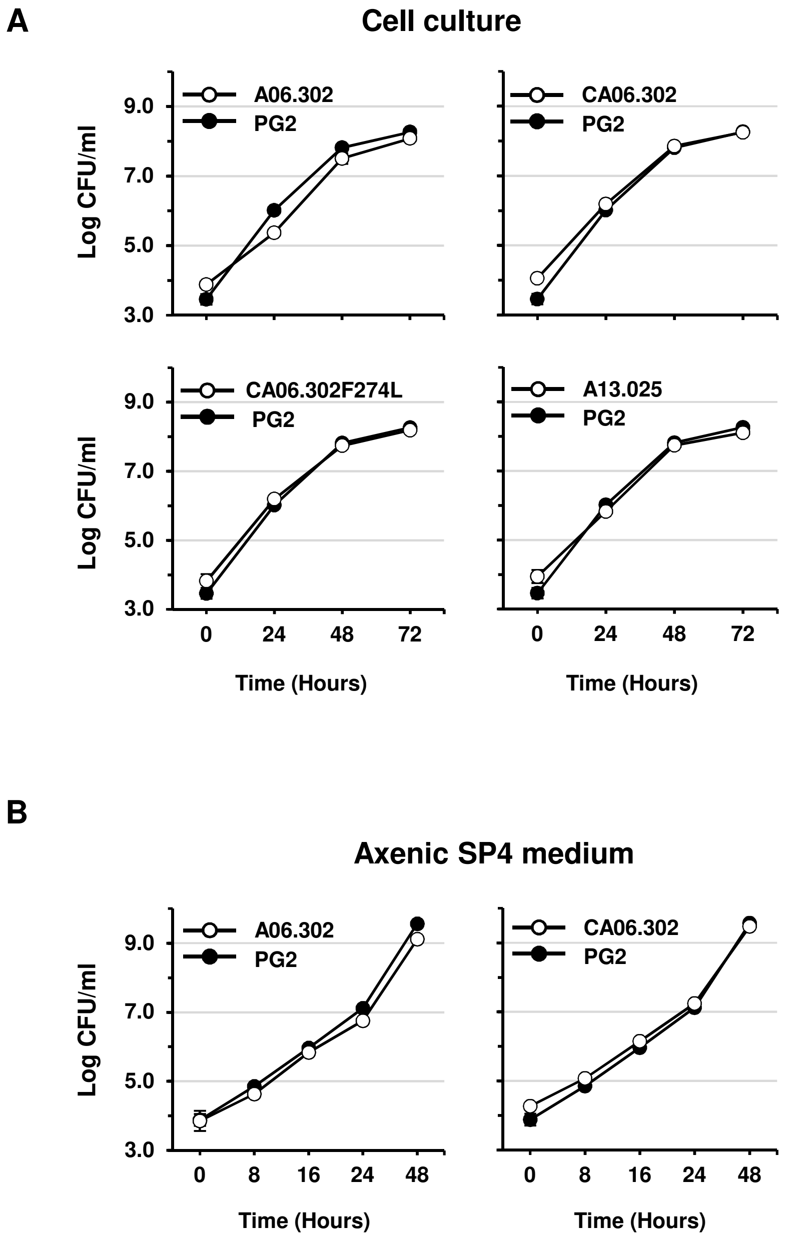

Figure S4. Growth curves of *p48* mutants and complemented strains under cell culture and axenic conditions. Mycoplasma growth was monitored under (A) cell culture and (B) axenic conditions. Changes in CFU titers of wild-type PG2 (closed circles) were compared with those of the *p48* mutant A06.302, the complemented strains CA06.302 and CA06.302F274, and the ABC transporter permease mutant A13.025. Data represent the mean of three independent experiments; error bars indicate standard deviations.

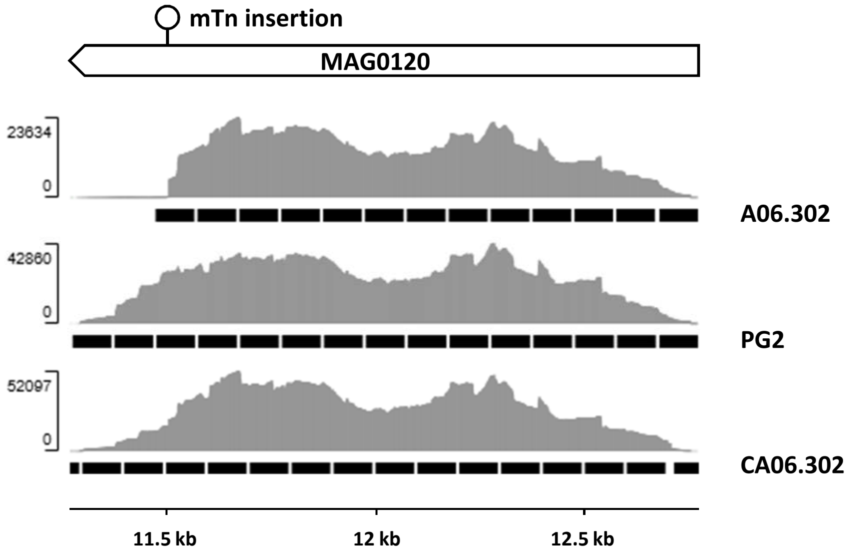

Figure S5. Detection of *p48* transcripts in the P48 mutant. RNA-seq detection of RNA transcripts from the *p48* gene (MAG0120) in the wild-type PG2 (PG2), the *p48* mutant A06.302 (A06.302) and the complemented strain CA06.302 (CA06.302) generated by transformation of the *p48* mutant A06.302 with plasmids pO/T-P48. The position of the transposon insertion site in MAG0120 is indicated (mTn). Y-axis shows read counts; black boxes denote gene regions with detected RNA transcripts.

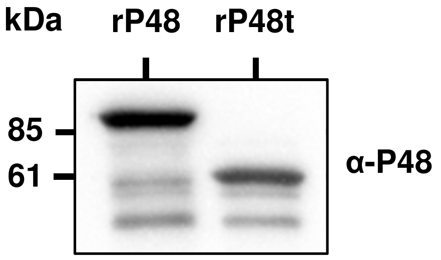

Figure S6. Reactivity of anti-P48 antibodies with a truncated recombinant P48 lipoprotein. Western blotting analysis of the reactivity of anti-P48 antibodies (α-P48) with full-length (rP48) and truncated forms of lipoprotein P48 (rP48t) expressed as recombinant soluble proteins in *E. coli.* The pH6HTN His_6-HaloTag® T7 vector (Promega) was used to express the soluble full-length (amino acid residues 26 to 465) and truncated (amino acid residues 26 to 246) P48 lipoprotein. Both proteins lack the N-terminal signal peptide (residues 1 to 25) and have an N-terminal His_6-HaloTag of approximately 35 kDa. Western blotting experiments were carried out using total protein extracts from *E. coli.*

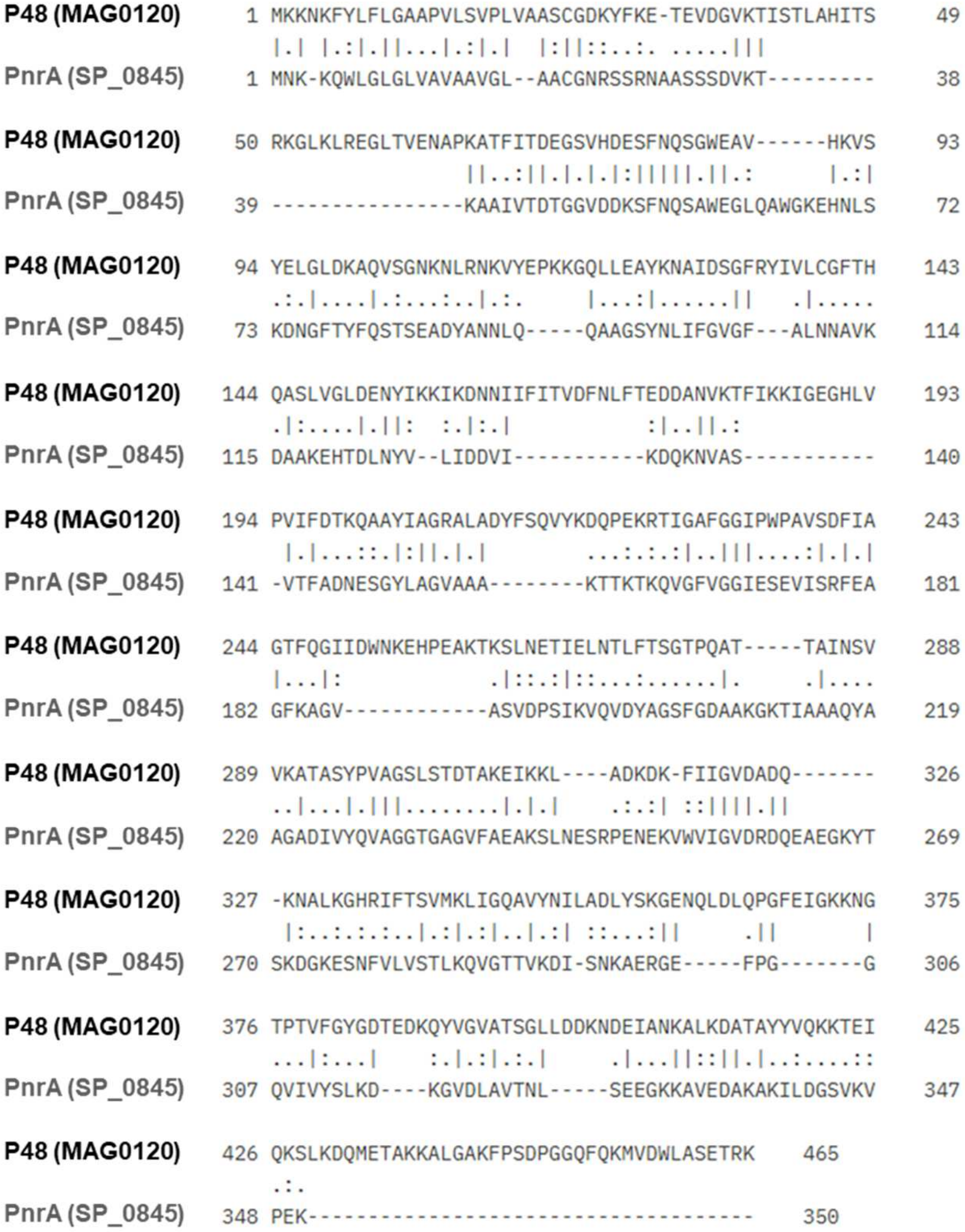

Figure S7. Sequence alignment of the *M. agalactiae* P48 and *S. pneumoniae* PnrA. Global sequence alignment of *Mycoplasma agalactiae* PG2 lipoprotein P48 (P48) and *Streptococcus pneumoniae* TIGR4 PnrA (PnrA) using EMBOSS Needle (https://www.ebi.ac.uk/jdispatcher/psa/emboss_needle). The *p48* and *pnrA* gene access numbers are indicated in parentheses.
