# Supplementary material for: Nucleoside binding by a surface lipoprotein governs conjugative ICE acquisition in mycoplasmas
Source: mBio. 2025 Dec 30;17(2):e02939-25. doi: 10.1128/mbio.02939-25 (PMC12892991; doi:10.1128/mbio.02939-25)
Supplement: Supplemental Tables — Tables S1 to S3. [file mbio.02939-25-s0002.docx]

**Table S1.** Top 50 DEGs in the *p48* mutant A06.302

| A06.302 vs PG2 ^a^ | | | | A06.302 vs CA06.302 ^a^ | | | | | |  |
| --- | --- | --- | --- | --- | --- | --- | --- | --- | --- | --- |
| Gene ^b^ | **FC ^c^ (Log_2_)** | **UniProtKB/TrEMBL ^d^** | | **Gene ^b^** | | **FC ^c^ (Log_2_)** | | **UniProtKB/TrEMBL ^d^** | |  |
| *Upregulated genes* | | | |  | | | | | |  |
| MAG1560 | **1.87** | **Uncharacterized lipoprotein** | | MAG2188 | | 2.38 | | No entry (tRNA-Phe) | |  |
| MAG4040 | **1.78** | **VirD4/TraG family (vICEA)** | | MAG7100 | | 2.00 | | Variable surface lipoprotein | |  |
| MAG0300 | 1.74 | Uncharacterized protein | | MAG0657 | | 1.74 | | No entry (tRNA-Arg) | |  |
| MAG1120 | 1.73 | No entry (pseudogene) | | MAG0700 | | 1.35 | | **Serine hydroxymethyltransferase** | |  |
| MAG4060 | **1.73** | **Uncharacterized protein (vICEA)** | | MAG0660 | | 1.30 | | ATPase, AAA family | |  |
| MAG0730 | 1.69 | Nitrogen fixation protein NifU | | **MAG1560** | | **1.29** | | **Uncharacterized lipoprotein** | |  |
| MAG4030 | **1.67** | **C5 Mtase (vICEA)** | | MAG2190 | | 1.28 | | Cytidine triphosphate synthase | |  |
| MAG0740 | 1.65 | DNA damage repair protein MucB | | **MAG4030** | | **1.24** | | **C5 Mtase (vICEA)** | |  |
| MAG2910 | 1.64 | Uncharacterized protein | | **MAG0710** | | **1.22** | | **Proline iminopeptidase** | |  |
| MAG4050 | 1.61 | Uncharacterized protein (vICEA) | | MAG0670 | | 1.19 | | Phenylalanine--tRNA ligase PheS | |  |
| MAG1140 | 1.60 | Uncharacterized protein | | **MAG4040** | | **1.12** | | **VirD4/TraG family (vICEA)** | |  |
| MAG4070 | 1.60 | Uncharacterized protein | | MAG0690 | | 1.11 | | Phenylalanine--tRNA ligase PheT | |  |
| MAG0720 | **1.60** | **Nitrogen fixation protein NifS** | | **MAG0310** | | **1.10** | | **Uncharacterized protein** | |  |
| MAG6150 | **1.50** | **No entry (pseudogene)** | | **MAG4060** | | **1.07** | | **Uncharacterized protein (vICEA)** | |  |
| MAG2900 | 1.49 | Uncharacterized protein | | MAG6780 | | 1.07 | | tRNA methyltransferase | |  |
| MAG6160 | 1.47 | No entry (pseudogene) | | MAG0680 | | 1.02 | | Uracil-DNA glycosylase | |  |
| MAG4010 | 1.45 | **IrrE N-term-like domain-containing protein** (vICEA) | | MAG6010 | | 0.98 | | Uncharacterized protein | |  |
| MAG3990 | 1.44 | **ssDNA-binding protein** (vICEA) | | **MAG0720** | | **0.96** | | **Nitrogen fixation protein NifS** | |  |
| MAG0310 | **1.39** | **Uncharacterized protein** | | MAG5030 | | 0.94 | | Lipoprotein P80 | |  |
| MAG0330 | 1.33 | Uncharacterized protein | | MAG6660 | | 0.90 | | Dephospho-CoA kinase | |  |
| MAG3900 | 1.29 | Uncharacterized protein (vICEA) | | MAG7110 | | 0.90 | | Integrase recombinase Xer | |  |
| MAG0710 | **1.27** | **Proline iminopeptidase** | | MAG0400 | | 0.86 | | Cysteinyl tRNA synthetase | |  |
| MAG2920 | 1.23 | Transmembrane protein | | MAG2240 | | 0.83 | | Holliday junction branch migration complex SU RuvB | |  |
| MAG6880 | 1.23 | Uncharacterized protein | | **MAG6150** | | 0.83 | | **No entry (pseudogene)** | |  |
| MAG3980 | 1.21 | Uncharacterized protein (vICEA) | | MAG2560 | | 0.83 | | Pseudogene | |  |
| *Downregulated genes* | | | |  | | | | | |  |
| MAG0550 | **-2.33** | | **Glyceraldehyde-3-phosphate dehydrogenase** | | MAG3190 | | -1.94 | | Enolase | |
| MAG2630 | **-1.78** | | **NADH oxidase** | | MAG2010 | | -1.90 | | Large ribosomal SU protein bL33B | |
| MAG1460 | -1.77 | | Chaperone protein DnaK | | **MAG0550** | | **-1.59** | | **Glyceraldehyde-3-phosphate dehydrogenase** | |
| MAG3510 | -1.74 | | ATP synthase subunit c | | MAG5880 | | -1.23 | | Transmembrane protein | |
| MAG7380 | **-1.63** | | **Thioredoxin** | | **MAG7380** | | **-1.20** | | **Thioredoxin** | |
| MAG2740 | -1.56 | | Alcohol dehydrogenase | | MAG4180 | | -1.07 | | Uridine phosphorylase | |
| MAG3500 | -1.52 | | ATP synthase A chain | | MAG4910 | | -1.05 | | Lipoyl-binding domain-containing protein | |
| MAG5110 | -1.47 | | Uridine phosphorylase | | MAG4560 | | -0.98 | | Small ribosomal SU protein uS9 | |
| MAG5870 | **-1.38** | | **Uncharacterized protein** | | **MAG5870** | | **-0.90** | | **Uncharacterized protein** | |
| MAG7250 | -1.37 | | Cytidylate kinase | | MAG0020 | | -0.88 | | DNA polymerase III, beta chain | |
| MAG0775 | -1.21 | | HTH HARE-type domain- containing protein | | MAG4680 | | -0.88 | | Acyl carrier protein phosphodiesterase | |
| MAG0100 | -1.17 | | Luciferase-like domain-containing protein | | MAG2350 | | -0.88 | | Variable surface lipoprotein | |
| MAG0160 | -1.15 | | Sugar ABC transporter permease | | **MAG0120** | | **-0.87** | | **Lipoprotein P48** | |
| MAG3960 | -1.15 | | **Uncharacterized protein** | | MAG6450 | | -0.87 | | Uncharacterized protein | |
| MAG3520 | -1.14 | | ATP synthase subunit b | | **MAG2630** | | **-0.85** | | **NADH oxidase** | |
| MAG4900 | -1.12 | | L lactate dehydrogenase | | MAG0010 | | -0.83 | | Chromosomal replication initiator protein DnaA | |
| MAG4280 | -1.12 | | Alcohol dehydrogenase | | MAG6180 | | -0.80 | | Large ribosomal SU protein bL12 | |
| MAG2220 | **-1.12** | | **Uncharacterized lipoprotein** | | MAG4530 | | -0.77 | | Ion trans domain protein | |
| MAG6370 | -1.11 | | Pentitol phosphotransferase enzyme II, B component | | MAG2320 | | -0.77 | | Glycerol ABC transporter permease gtsB | |
| MAG0120 | **-1.10** | | **Lipoprotein P48** | | **MAG2220** | | **-0.76** | | **Uncharacterized lipoprotein** | |
| MAG4690 | -1.10 | | Phosphocarrier protein HPr | | MAG5140 | | -0.75 | | Triosephosphate isomerase | |
| MAG5120 | -1.06 | | Thymidine phosphorylase | | MAG2330 | | -0.75 | | Glycerol ABC transporter permease gtcC | |
| MAG3540 | -1.06 | | ATP synthase subunit alpha | | MAG4190 | | -0.73 | | **Uncharacterized protein** | |
| MAG3460 | -1.06 | | **Uncharacterized protein** | | MAG4970 | | -0.71 | | Hexosephosphate transport protein | |
| MAG3050 | -1.06 | | **Uncharacterized protein** | | MAG6400 | | -0.70 | | **Uncharacterized protein** | |

^a^ RNA sequencing was performed on three biological replicates; DEG with a p-value < 0.01 and adjusted p-value < 0.01; CA06.302 designates the complemented derivative of the *p48* mutant A06.302; entries identified in both comparative analysis are in bold letters; vICEA: genes associated with the vestigial ICEA identified in the PG2 genome (18); ^b^ CDS identifier from the PG2 genome in the GenBank database ([CU179680.1](https://www.ncbi.nlm.nih.gov/nuccore/148291314)); ^c^ Log_2_ Fold Change (FC) in RNA levels; ^d^ Protein names according to UniProtKB/TrEMBL entries in the UniProt Knowledgebase (UniProtKB; <https://www.uniprot.org/>).

**Table S2.** Top 10 proteins immunoprecipitated with anti-P48 and anti-CDS14 antibodies ^a^

| **Proteins ^b^** | **CDS ^c^** | **IBAQ (%) ^d^** |
| --- | --- | --- |
| ***Proteins co-IP with P48 antibodies*** |  |  |
| **Variable surface lipoprotein Y** | **MAG7080** | **12.86** |
| **Variable surface lipoprotein** | **MAG3600** | **7.14** |
| **Variable surface lipoprotein X** | **MAG7070** | **5.90** |
| **Lipoprotein P48** | **MAG0120** | **3.92** |
| **LemA family protein** | **MAG1220** | **3.67** |
| **Small ribosomal subunit protein uS3** | **MAG5400** | **3.12** |
| **NADH oxidase** | **MAG2630** | **2.78** |
| ATP-dependent zinc metalloprotease | MAG7450 | 2.39 |
| **Lipoprotein P80** | **MAG5030** | **2.26** |
| **Hexosephosphate transport protein** | **MAG4970** | **2.15** |
| ***Protein co-IP with CDS14 antibodies*** |  |  |
| Lipoprotein CDS14 | cds14 | 14.32 |
| **Variable surface lipoprotein Y** | **MAG7080** | **11.55** |
| **Variable surface lipoprotein X** | **MAG7070** | **8.47** |
| **Variable surface lipoprotein** | **MAG3600** | **7.49** |
| **NADH oxidase** | **MAG2630** | **2.75** |
| **Lipoprotein P48** | **MAG0120** | **2.46** |
| **Hexosephosphate transport protein** | **MAG4970** | **2.34** |
| **Lipoprotein** | **MAG6200** | **2.17** |
| **Small ribosomal subunit protein uS3** | **MAG5400** | **1.70** |
| **Lipoprotein P80** | **MAG5030** | **1.49** |

^a^ Proteomic analysis of the anti-CDS14 and anti-P48 eluates following co-incubation of PG2^T^[ICEA]^G^ (ICEA donor) and pPG2^P^ (recipient); ^b^ Protein names according to UniProtKB/TrEMBL entries in the UniProt Knowledgebase (UniProtKB; <https://www.uniprot.org/>); ^c^ Accession number of the CDS from the PG2 genome and ICEA in the GenBank database (accession number: [CU179680.1](https://www.ncbi.nlm.nih.gov/nuccore/CU179680.1) and [CT030003.1](https://www.ncbi.nlm.nih.gov/nuccore/CT030003), respectively); ^d^ **Intensity-Based Absolute Quantification values**.

**Table S3.** Oligonucleotide primers

| **Application** | **Primer name** | **Sequence (5’ -> 3’) ^a^** |
| --- | --- | --- |
| Single-primer PCR amplification and sequencing of genomic DNA from *M. agalactiae* and *M. bovis* mutants | SG6_2 | GCCGCGTCAATTGGTGGGGT |
|  | M13-24b_Rev | AGCTGTTTCCTGTGTGAAATTGTT |
|  | M13-30bases_REV | GGTCATAGCTGTTTCCTGTGTGAAATTGTT |
|  | SG5 | TTTTACACAATTATACGGACTTTATC |
| Cloning of the PG2 *p40* promoter | p40RF-CC | ACGGGGCTAAAGAAGCTGAT |
|  | P40/P48 N-ter | CTAAAAATAGATAGAATTTATTTTTCTTCATAATTATTTATATCCTTTTC |
| Cloning and mutagenesis of PG2 MAG0120 (*p48*) | P48 C-ter | CTACAGTAAGTAATGACTCGACCA |
|  | P48A1204T F | ACATCAGGATTATTAGATGATTAAAATGATGAAATAGCTAAC |
|  | P48A1204T R | CATCATTTTAATCATCTAATAATCCTGATGTTG |
|  | P48C739T F | TAGCTGGTACTTTCTAAGGTATTATTGACTG |
|  | P48C739T R | AATACCTTAGAAAGTACCAGCTATAAAGTCAG |
|  | T822AF | GAGCTAAATACTTTATTAACTTCAGGAACACCTCAA |
|  | T822AR | TTGAGGTGTTCCTGAAGTTAATAAAGTATTTAGCTC |
| Cloning of the 5632 MAGa0140 (*p48* homolog) | P40/P48 N-ter | CTAAAAATAGATAGAATTTATTTTTCTTCATAATTATTTATATCCTTTTC |
|  | P48 C-ter 5632 | ACGCTACAGTAAGTAATCAC |
| Cloning of the RM16 FWK06_000085 (*p48* homolog) | P40/P48 N-ter RM16 | CTAAAAATAGATAGAATTTGTTTTTTTTCATAATTATTTATATCCTTTTC |
|  | P48 C-ter RM16 | GTAATGAAGCAACTAAAAAAG |
| PCR detection of ICEA CDS22 | ORF22-F-ICE | TGAGACCAGCAAGCTGAAGA |
|  | ORF22-R-ICE | TCTGTATCAATCTGAATTGCATCAT |
| PCR amplification of ICEA integration site in PG2^T^[ICEA]^G^ | ICE MAG6030 F | AGGCTAAAAGTGCTTTGGAG |
|  | ICE MAG6030 R | GCTTTGAGTGAAATTGAGAGTCA |
| PCR detection of the *p48* gene | P48-F | GCAGCTTGTTTAGTGTCAAAG |
|  | P48-R | CCTAAAGCAACCTTTATAACTG |
| PCR detection of the G-Tag | GM1 | ACATGAATTACACGAGGGC |
|  | GM2 | GTTCTTCTTCTGACATAGTAG |
| PCR detection of the P-Tag | PuroF2 | GTTGCTGTTTGGACTACTCCAG |
|  | PuroR | CACCAAGTTCTAGGACCTTCAGG |
| PCR amplification of mTn insertion site in *p48* mutant A06.302 | Seq_P48 F3 | GCTGACTTATATAGCAAAGGTGAAA |
|  | P48 C-term | CTACAGTAAGTAATGACTCGACCA |

**^a^** Underlined nucleotides indicate single nucleotide substitutions
