# Supplementary figures and images for: Nucleoside binding by a surface lipoprotein governs conjugative ICE acquisition in mycoplasmas
Source: mBio. 2025 Dec 30;17(2):e02939-25. doi: 10.1128/mbio.02939-25 (PMC12892991; doi:10.1128/mbio.02939-25)

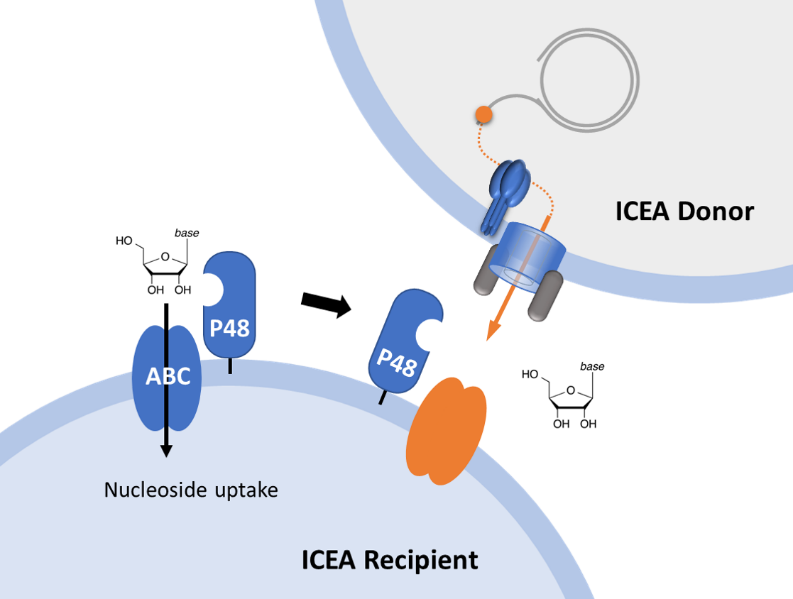

Supplement: Graphical abstract — Surface nucleoside sensing by P48 controls ICEA acquisition in mycoplasmas: P48 promotes ICEA transfer by stabilizing donor–recipient interactions, potentially coupling donor ICE machinery with recipient competence-like functions, and/or by sensing extracellular nucleoside availability to modulate cellular states permissive for ICE integration. [file mbio.02939-25-s0003.tif]
